# ﻿Taxonomic treatment on *Garciniasopsopia* (Section Brindonia, Clusiaceae) in Thailand, with a new synonym and three lectotypifications of its synonyms

**DOI:** 10.3897/phytokeys.254.147960

**Published:** 2025-03-26

**Authors:** Chatchai Ngernsaengsaruay, Pichet Chanton, Weereesa Boonthasak, Nittaya Mianmit, Tharnrat Kaewgrajang

**Affiliations:** 1 Department of Botany, Faculty of Science, Kasetsart University, Chatuchak, Bangkok 10900, Thailand Kasetsart University Bangkok Thailand; 2 Biodiversity Center, Kasetsart University (BDCKU), Chatuchak, Bangkok 10900, Thailand Suan Luang Rama IX Foundation, Nong Bon Subdistrict Bangkok Thailand; 3 Suan Luang Rama IX Foundation, Nong Bon Subdistrict, Prawet District, Bangkok, 10250, Thailand Highland Research and Development Institute Chiang Mai Thailand; 4 Royal Park Rajapruek, Highland Research and Development Institute (Public Organization), Chiang Mai 50100, Thailand Kasetsart University Bangkok Thailand; 5 Department of Forest Management, Faculty of Forestry, Kasetsart University, Chatuchak, Bangkok, 10900, Thailand Suan Luang Rama IX Foundation, Nong Bon Subdistrict Bangkok Thailand; 6 Department of Forest Biology, Faculty of Forestry, Kasetsart University, Chatuchak, Bangkok, 10900, Thailand Highland Research and Development Institute Chiang Mai Thailand

**Keywords:** Dioecy, exudate containing canals, glandular wavy lines, Guttiferae, lectoty­pifications, Malpighiales, synonymisation, taxonomy

## Abstract

*Garciniasopsopia* belongs to the section Brindonia in the family Clusiaceae. The fruits, young shoots and leaves are edible and have a sour taste. Morphological description and illustrations are provided, along with notes on distribution, habitats and ecology, phenology, a preliminary conservation assessment, etymology, vernacular names, uses and specimens examined. *Garciniamckeaniana* is a newly-synonymised name under *G.sopsopia*. Three synonyms of *G.sopsopia* are here lectotypified, including *G.paniculata*, *G.rhumicowa* and *G.mckeaniana*.

## ﻿Introduction

*Garcinia* L. is a group of evergreen trees, occasionally shrubs, which are usually dioecious, but sometimes polygamo-dioecious. It also has obligately and facultatively agamospermous species (Ngernsaengsaruay et al. 2023a). The genus comprises approximately 400 species (Gaudeul et al. 2024; POWO 2025) and is the largest genus in the Clusiaceae Lindl. (Guttiferae Juss.). It is a pantropically distributed genus and has centres of diversity located in Africa (Madagascar), Australasia and Southeast Asia (Sweeney and Rogers 2008; Gaudeul et al. 2024). In Asia, *Garcinia* is most diverse in the Malesian Region, but also spreads north into southern China, west to India and east to the Micronesian islands (Nazre et al. 2018).

In Thailand, the genus *Garcinia* was enumerated by Craib (1925), with 20 species. Gardner recorded six species in northern Thailand (Gardner et al. 2000) and 23 species (including five unidentified species) in Peninsular Thailand (Gardner et al. 2015). A taxonomic revision of *Garcinia* in Thailand has recently been undertaken by the first author as part of the Flora of Thailand project. Ngernsaengsaruay and Suddee (2016, 2022) described new species, *G.nuntasaenii* Ngerns. & Suddee from north-eastern and *G.santisukiana* Ngerns. & Suddee from eastern Thailand, respectively. Ngernsaengsaruay (2022) recognised three species in G.sectionBrindonia (Thouars) Choisy in Thailand, i.e. *G.atroviridis* Griff. ex T. Anderson, *G.lanceifolia* Roxb. and *G.pedunculata* Roxb. ex Buch.-Ham. Ngernsaengsaruay et al. (2022a, 2023a) published additional new species records from Peninsular Thailand, *G.dumosa* King and *G.exigua* Nazre, respectively. Ngernsaengsaruay et al. (2022b) described *G.siripatanadilokii* Ngerns., Meeprom, Boonthasak, Chamch. & Sinbumr. as a new species from Peninsular Thailand. GarciniasectionXanthochymus (Roxb.) Pierre was revised for Thailand, with four native species: *G.dulcis* (Roxb.) Kurz, *G.nervosa* (Miq.) Miq., *G.prainiana* King and *G.xanthochymus* Hook. f. ex T. Anderson (Ngernsaengsaruay et al. 2023b). GarciniasectionGarcinia L. was treated for Thailand, with three species and one variety, i.e. two native species: *G.celebica* L. and *G.exigua* and one cultivated species: G.mangostanaL.var.mangostana, including excluded and unplaced species, *G.anomala* Planch. & Triana (Ngernsaengsaruay et al. 2024a). Ngernsaengsaruay et al. (2024b) published an additional new species record from Peninsular Thailand, *G.minutiflora* Ridl. Finally, *Garcinia* sections *Dicrananthera* Pierre and *Macrostigma* Pierre were revised for Thailand. Three species were enumerated, i.e. one species, *G.thorelii* Pierre, belongs to the section Dicrananthera and two species, *G.nuntasaenii* and *G.prainiana*, are in the section Macrostigma (Ngernsaengsaruay and Chanton 2024). The sectional level taxonomy in *Garcinia* was recently updated by Gaudeul et al. (2024) and Sweeney and Gaudeul (2024).

*Oxycarpussopsopia* Buch.-Ham. was described in 1826 (Buchanan-Hamilton 1826) and transferred to the genus *Garcinia* by Mabberley (1977). Mabberley (1977) selected the syntype *F. Buchanan-Hamilton 1120* housed at E [E00438015] collected at Goalpara, India, “habitat in sylvis Camrupae orientalis” as the lectotype. The same author synonymised *Garciniapaniculata* (G. Don) Roxb. under *G.sopsopia*. *Garciniasopsopia* belongs to the section Brindonia (Jones 1980; Gaudeul et al. 2024). *Garciniamckeaniana* was described by William Grant Craib based on the syntypes, *A. F. G. Kerr 3470* and *A. F. G. Kerr 3504* collected in Doi Suthep, Thailand, at elevations of 1,200–1,550 m a.m.s.l. (Craib 1924).

We examined the protologues, types and general specimens of *Garciniasopsopia* and *G.mckeaniana* and found that *G.mckeaniana* shares vegetative and reproductive characters with *G.sopsopia*. *Garciniasopsopia* (basionym: *Oxycarpussopsopia*) is the earliest name for the species. Therefore, *G.mckeaniana* is treated here as a new synonym of *G.sopsopia*.

In this paper, we provide a taxonomic treatment on *Garciniasopsopia* in Thailand that includes synonymisation, lectotypifications, a detailed morphological description and illustrations, along with notes on distribution, habitats and ecology, phenology, a preliminary conservation assessment, etymology, vernacular names, uses and specimens examined.

## ﻿Materials and methods

The collected specimens were examined by consulting taxonomic literature (e.g. Anderson (1874); Kurz (1877); Pierre (1883); Vesque (1889, 1893); Engler (1893); Craib (1924); Kanjilal et al. (1934); Gagnepain (1943); Maheshwari (1964); Mabberley (1977); Jones (1980); Long (1984); Singh (1993) and by comparing with herbarium specimens housed in the following herbaria: AAU, BKF, BM, C, CMUB, K, P, QBG and those included in the virtual herbarium databases of A, GH, AAU, BM, BR, CAL, E, G, K, L, P, The Wallich Catalogue Online and MICH (from GBIF, https://www.gbif.org/). All herbarium codes follow Thiers (2024, continuously updated). All specimens cited have been seen by the authors unless stated otherwise. The taxonomic history of the species was compiled using the literature and online databases (IPNI 2025; POWO 2025). The morphological characters, distribution, habitats and ecology, phenology and uses were described from historic and newly-collected herbarium specimens and the author’s observations during fieldwork. The vernacular names were compiled from the specimens examined and literature (e.g. Pooma and Suddee (2014)). Thailand floristic regions follow *Flora of Thailand* Vol. 4(3.3) (The Forest Herbarium, Department of National Parks, Wildlife and Plant Conservation 2023). The preliminary assessment of conservation status was performed following the IUCN Red List Categories and Criteria (IUCN Standards and Petitions Committee 2024) combined with GeoCAT analysis (Bachman et al. 2011) and field information.

## ﻿Results and discussion

### ﻿Taxonomic treatment

#### ﻿ Garcinia sopsopia

Taxon classificationPlantaeMalpighialesClusiaceae

(Buch.-Ham.) Mabb., Taxon 26(5–6): 529. 1977.

36AA5251-A5FD-56A4-89BF-A5A8E66AFFAD

Figs 1 , 2 , 3

≡ Oxycarpussopsopia Buch.-Ham., Mem. Wern. Nat. Hist. Soc. 5(2): 345. 1826.Type. lectotype (designated by Mabberley (1977)), India, Assam, Goalpara, “habitat in sylvis Camrupae orientalis”, 1 Jun 1808, *F. Buchanan-Hamilton 1120*, E image! [E00438015]. Fig. 4A.
= Stalagmitispaniculata G. Don, Gen. Hist. 1: 621. 1831.
= Garciniapaniculata (G. Don) Roxb., Fl. Ind. 2: 626. 1832.
= Garciniabobee-cowa Choisy, Descr. Guttif. Inde: 35. 1849.
= Stalagmitisboobicowa G. Don, Gen. Hist. 1: 621. 1831, nom. nud. Type. lectotype (designated here), India, cultivated in Calcutta Botanical Garden (H.B.C.) (originally from Sylhet, Bangladesh), ♂ fl., s.d., *Wallich Cat. 4857B*, G image! [G00726286]; isolectotypes: CAL image! [CAL0000065167]; isolectotype: K! [K001104077]. Fig. 4B.
= Garciniarhumicowa Choisy, Descr. Guttif. Inde: 35. 1849. Type. lectotype (designated here), Bangladesh, Sylhet, ♂ fl., s.d., *F. De Silva*, *Wallich Cat. 4858B*, G image! [G00726295]; isolectotypes: BR image! [BR0000036486748], CAL image! [CAL0000065165], K! [K000677604, K001104080], P images! [P04701880, P04701886] (cited as “Garciniabhumicowa Roxb.” on the label, as a nom. nud.). Fig. 5A.
= Garciniamckeaniana Craib, Bull. Misc. Inform. Kew 1924(3): 84. 1924. Type. lectotype (designated here), Thailand, Chiang Mai, Doi Suthep, ♂ fl., *A. F. G. Kerr 3470*, K! [K000677701]; isolectotypes: BM image! [BM000611632], P! [P05061534]), syn. nov. Fig. 5B.

##### Description.

***Habit*** evergreen trees, dioecious, 8–20 m tall, 50–120(–150) cm gbh; exudate pale yellow, sticky; branches decussate, horizontal or nearly horizontal; young branchlets green, 4-angular to slightly 4-angular, glabrous. ***Bark*** brown or reddish-brown, smooth or slightly rough; inner bark red or reddish-pink. ***Terminal bud*** concealed between the bases of the uppermost pair of petioles. ***Leaves*** decussate; lamina elliptic, elliptic-oblong, narrowly elliptic or oblanceolate-obovate, 9.5–23 × 4–10.5 cm, apex acuminate or acute, base cuneate, sometimes obtuse, margin entire or repand, subcoriaceous, slightly bullate, dark green above, paler below, glabrous and shiny on both surfaces, midrib slightly raised (proximal part) and flattened (distal part) above, raised below, secondary veins 8–12 each side, 0.7–2 cm apart from each other, curving towards the margin and connected in distinct loops and united into an intramarginal vein, flattened above, raised below, intersecondary veins usually absent, tertiary veins scalariform, veinlets reticulate, visible on both surfaces, with scattered brown gland dots on both surfaces, interrupted long wavy lines (glandular wavy lines, also called exudate containing canals) present, of differing lengths, running across the secondary veins to the apex, visible on both surfaces especially on the lower surface of dry leaves; petiole green, 0.9–1.8 cm long, 1.2–4 mm diam., not grooved, glabrous, with a basal appendage clasping the branchlet; in fresh leaves, brittle when crushed; in young leaves, brownish-red, turning pale green, glossy. ***Inflorescences*** terminal, a thyrse with many to numerous flowers, 4–12 cm long, glabrous; bracts early caducous, triangular, 1–1.8 × 1–1.7 mm; peduncle 1.2–2.8 cm long, 1–3 mm diam., 4-angular; rachis 3.6–8 cm long, 1–2.7 mm diam., 4-angular. ***Flowers*** unisexual, 4-merous; bracteoles early caducous; sepals and petals decussate, concave, glabrous. ***Flower buds*** green, subglobose to globose, 2.8–5 mm diam. ***Staminate flowers*** in a much-branched thyrse (3.5–11.5 cm wide), with decussate branches, fully open flowers 0.9–1.6 cm diam.; pedicel green, 1.7–3.8 mm long, 0.5–2 mm diam., 4-angular; sepals 4, green, thinly coriaceous; outer sepals broadly ovate or ovate, 1.8–3 × 1–2 mm, apex rounded; inner sepals broadly elliptic, elliptic or suborbicular, 2–3.2 × 1.3–2.3 mm, apex rounded; the outer pair slightly smaller than the inner pair; petals 4, pale yellow to yellow, slightly thick and fleshy, oblong, 3–5.8 × 2.6–4.7 mm, subequal (thicker and longer than sepals), apex rounded, gradually reflexed after anthesis; stamens numerous, united into a single central 4-sided or weakly 4-lobed bundle surrounding a pistillode, bundle 3–4 × 2.6–3.8 mm; filaments very short; anthers 4-thecous, small, longitudinally dehiscent; pistillode creamish-white, mushroom-shaped, 1.3–2.7 mm long; rudimentary ovary small; sterile stigma, sessile, slightly convex, radiate, shallowly 5–7-lobed, 0.5–1 mm diam., papillate. ***Pistillate flowers*** in a short-branched thyrse, fully open flowers same as or slightly larger than staminate flowers; pedicel green, short and thick (slightly shorter and thicker than in staminate flowers), 4-angular; sepals and petals same as or slightly larger than in staminate flowers; staminodes absent; pistil mushroom-shaped, ovary globose or subglobose, 2–3 mm diam., glabrous, 5–7-locular; stigma sessile, convex, radiate, shallowly 5–7-lobed, papillate. ***Fruits*** berries, green, turning bright yellow, when ripe, glabrous and glaucous, cut fruits with a sticky yellow exudate, globose or subglobose, 4.5–7 × 4.3–6.3 cm, sometimes oblique, asymmetrical, without or with a short, thick beak and concave at the apex, with 6–8 longitudinal sutures, pericarp fleshy, 0.7–1.2 cm thick; persistent stigma dark brown or blackish-brown, 2.5–4 mm diam., indistinctly lobed, papillate; persistent sepals 2–4.5 × 3–5.7 mm, larger than in flowering material; fruiting stalk short and thick, 3–4.5 mm long, 5–7 mm diam. ***Seeds*** 3–7, sometimes aborted (1–2), dark brown mottled with paler irregular lines, semi-ellipsoid, 1.8–3 × 0.8–1.2 cm, rounded at both ends, with a yellow fleshy pulp.

**Figure 1.**
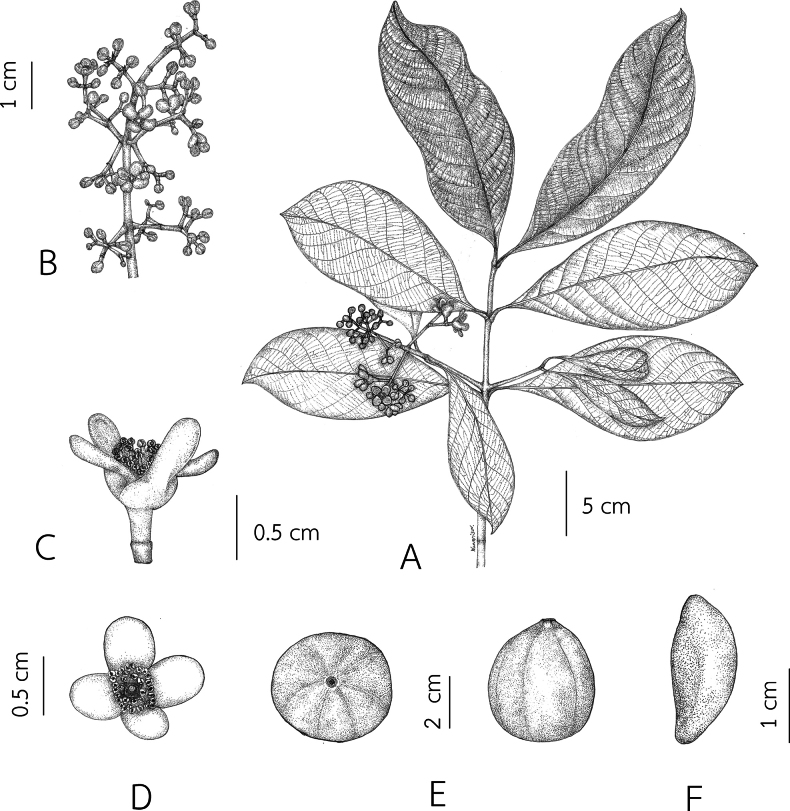
*Garciniasopsopia***A** branchlets, leaves and staminate inflorescences with flower buds and open flowers **B** staminate inflorescences with flower buds and open flowers **C, D** fully open staminate flowers **E** fruits **F** seed. Drawn by Wanwisa Bhuchaisri.

**Figure 2.**
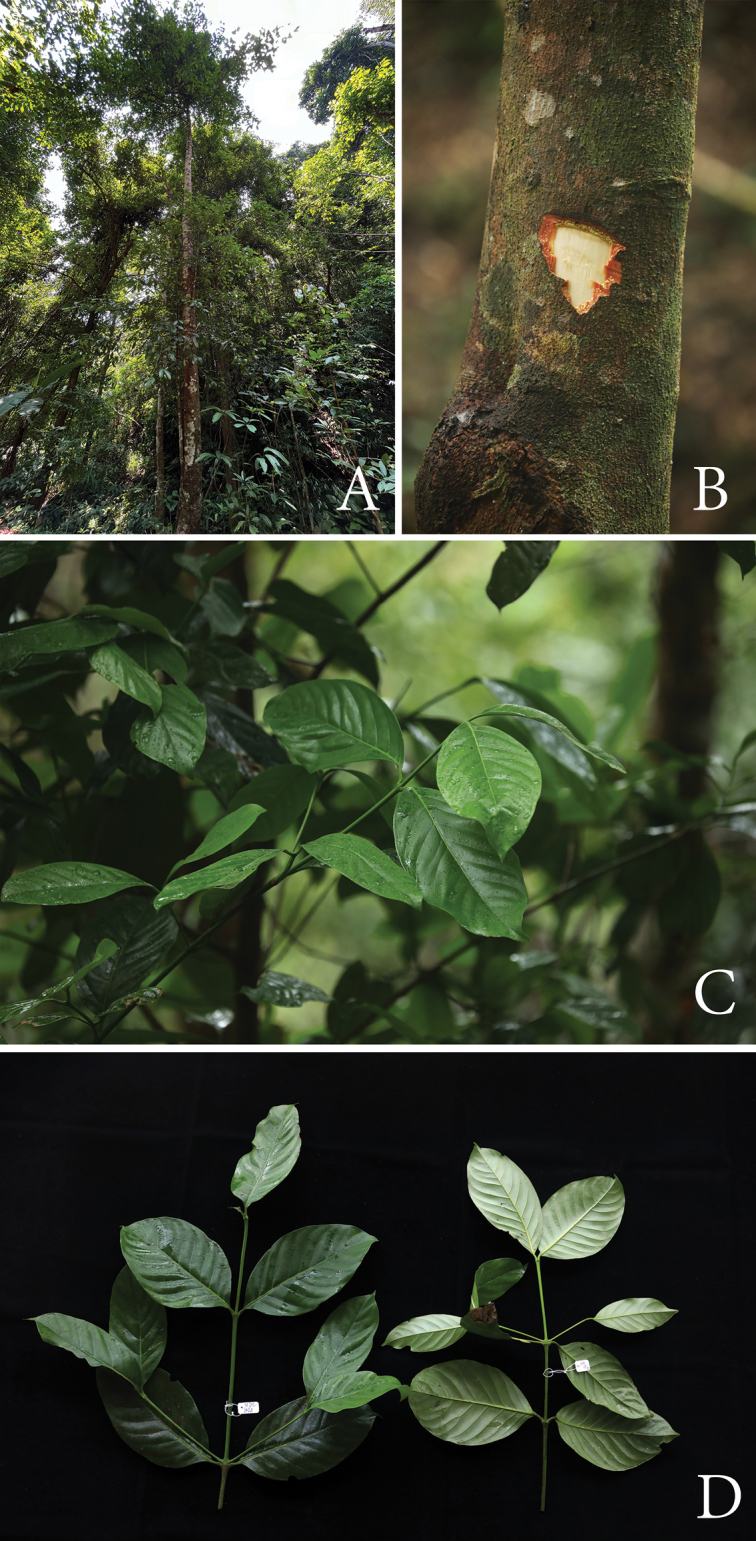
*Garciniasopsopia***A** habit and habitat **B** slashed bark with yellow exudate **C** branchlets and leaves **D** branchlets and leaves: upper leaf surfaces (left) and lower leaf surfaces (right). Photos: Chatchai Ngernsaengsaruay.

**Figure 3.**
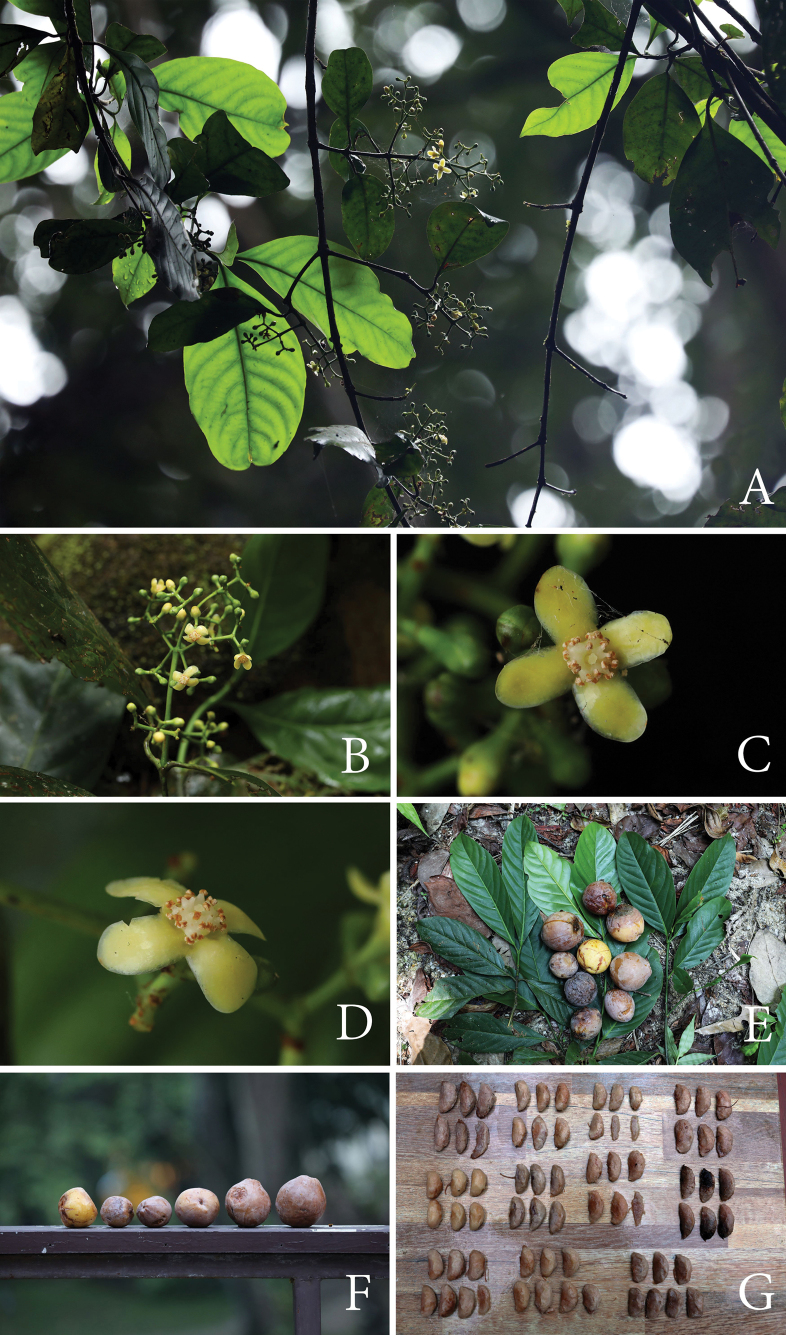
*Garciniasopsopia***A** branchlets, leaves and staminate inflorescences with flower buds and open flowers **B** staminate inflorescences with flower buds and open flowers **C, D** fully open staminate flowers **E** branchlets, leaves and ripe fruits **F** ripe fruits **G** seeds. Photos: Chatchai Ngernsaengsaruay.

**Figure 4.**
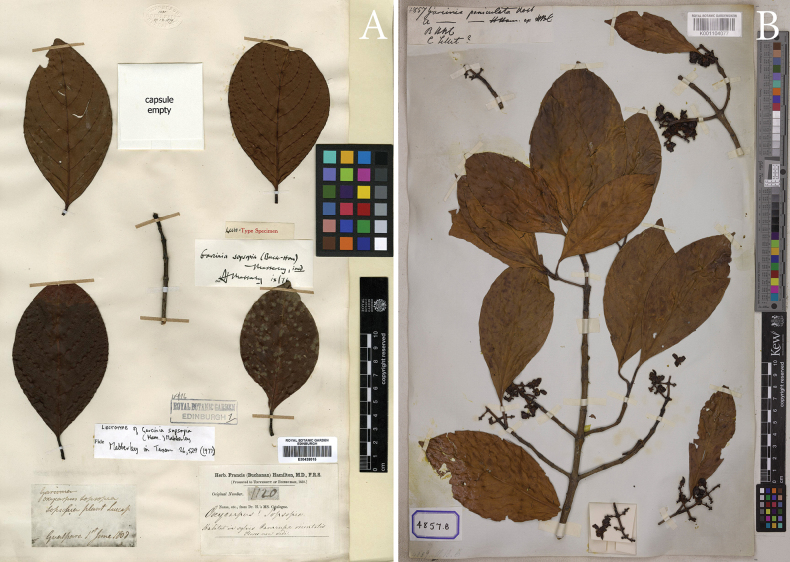
Lectotype of *Garciniasopsopia* and isolectotype of *Garciniapaniculata***A***Garciniasopsopia*, *F. Buchanan-Hamilton 1120* (E00438015) from Goalpara, “habitat in sylvis Camrupae orientalis”, Assam, India, lectotype selected by Mabberley (1977)**B***Garciniapaniculata*, a synonym of *Garciniasopsopia*, *Wallich Cat. 4857B* (K001104077) cultivated in Calcutta Botanical Garden (H.B.C.), India (originally from Sylhet, Bangladesh), isolectotype selected here. Photos: © 2018 Royal Botanic Garden Edinburgh, https://data.rbge.org.uk/herb/E00438015 (**A**), © The Board of Trustees of the RBG, Kew (**B**).

**Figure 5.**
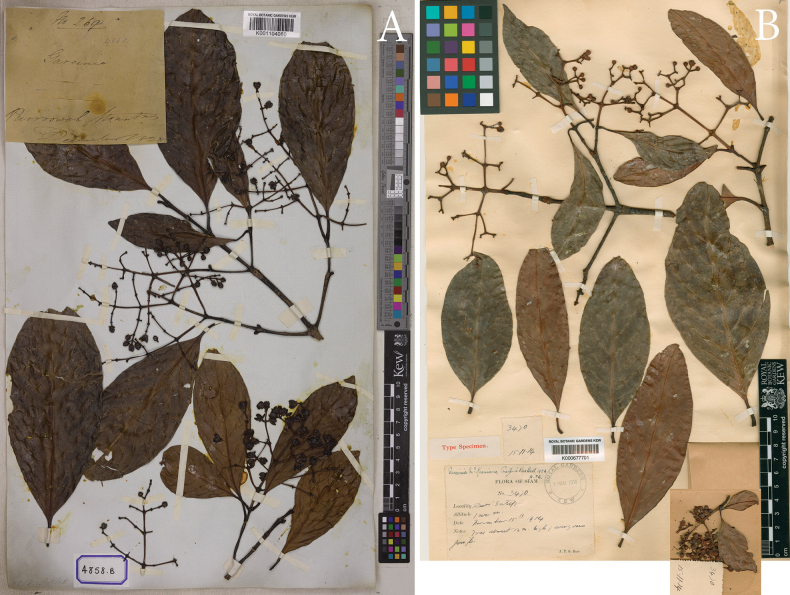
Isolectotype of *Garciniarhumicowa* and lectotype of *Garciniamckeaniana***A***Garciniarhumicowa*, a synonym of *Garciniasopsopia*, *F. De Silva, Wallich Cat. 4858B* (K001104080) from Sylhet, Bangladesh (cited as “*Garciniabhumicowa* Roxb.” on the label, as a nom. nud.), isolectotype selected here **B***Garciniamckeaniana*, a new synonym of *Garciniasopsopia*, *A. F. G. Kerr 3470* (K000677701) from Doi Suthep, Chiang Mai Province, Thailand, lectotype selected here. Photos: © The Board of Trustees of the RBG, Kew.

##### Distribution.

**Figure 6.**
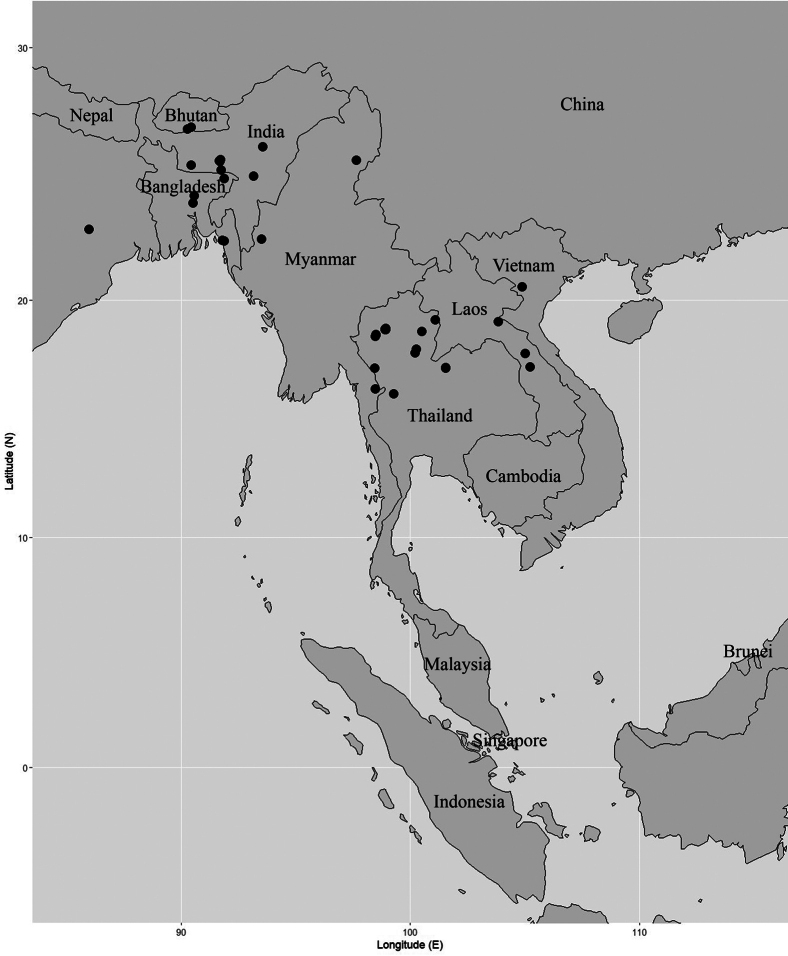
Distribution of *Garciniasopsopia*. It is known from India (Assam, Meghalaya), Nepal to Myanmar and Vietnam. In Thailand, this species is known to be naturally distributed in the northern and the north-eastern regions. Photo: Pichet Chanton and Chatchai Ngernsaengsaruay.

India (Assam, Meghalaya), Nepal, Bhutan, Bangladesh, Myanmar, Vietnam, Laos, Thailand. The distribution record of *Garciniasopsopia* was published without coordinates, but it includes a textual description of its location (Fig. 6).

##### Distribution in Thailand.

**Northern**: Chiang Mai, Nan, Phrae, Uttaradit, Tak, Kamphaeng Phet; **North-eastern**: Loei. Fig. 6.

##### Habitat and ecology.

This species is found in lower montane rain forests, lower montane pine-oak forests and dry evergreen forests, sometimes along streams, at elevations of 500–1,550 m a.m.s.l.

##### Phenology.

Flowering in September to January; fruiting in March to June (August).

##### Conservation status.

*Garciniasopsopia* is widely distributed from India, Nepal to Myanmar and Vietnam. It is known from many localities and has a large EOO of 1,779,647.52 km^2^ and an AOO of 128 km^2^. In Thailand, this species is known to be naturally distributed in the northern and the north-eastern regions and has an EOO 79,178.24 km^2^ and an AOO of 56 km^2^. Therefore, we consider the conservation assessment here as Least Concern (LC).

##### Etymology.

The specific epithet of *Garciniasopsopia* is derived from “Sopsopiya Bengalensium” (Buchanan-Hamilton 1826; Mabberley 1977). The specific epithet of *G.paniculata* is a Latin word meaning with a branched-racemose or cymose inflorescence (Gledhill 2002). However, from our examination of specimens, the staminate inflorescence of this species is a terminal, many-branched thyrses and the pistillate inflorescence is a terminal, short-branched thyrses. The specific epithet of *G.mckeaniana* honors James W. McKean, MD (1860–1949). He was an American doctor and missionary who pioneered leprosy work in Thailand, including the construction of the Chiang Mai Leprosarium in 1908. He began his work in Chiang Mai in 1889 and remained there for his entire missionary life, carrying out general medical and evangelical work (https://leprosyhistory.org/database/person99).

##### Vernacular names.

**Ma da** (มะดะ) (Uttaradit, from the specimen *P. Kanchanapan 30*); Boobee-Kowa, Bubi Kowa (India); Sochopa-tenga, Sosopatenga (Assam); Sopsopia garcinia, Sopsop garcinia (English).

##### Uses.

The fruits (pericarp and fleshy pulp surrounding the seeds), young shoots and leaves are edible and have a sour taste. In India, it is often cultivated for its edible fruits and the leaves are also said to be edible (Jones 1980; Singh 1993). In Assam, the ripe fruits are eaten raw (Baruah et al. 2021), the fleshy pulp being used for making refreshing drinks (Brahma et al. 2022). The leaves are used to treat roundworm (Baruah et al. 2021). Moderately hard wood is used for house construction and firewood (Baruah et al. 2021). In Vietnam, five xanthones were isolated and identified from *Garciniasopsopia* for the first time. Garcinone E and bannanxanthone E displayed a significant inhibitory effect against the growth of bacterium *Staphylococcusaureus* (Nguyen et al. 2020).

##### Lectotypifications.

*Garciniapaniculata* was named by William Roxburgh, found in a few gardens about Calcutta, was originally from Sylhet (“Silhet” or “Sillet”), where the species is indigenous and known to the natives by the name Boobee-Kowa (Roxburgh 1832). He did not choose a holotype nor did he mention the collector number and the name of the herbaria where the specimen was housed. He also did not provide a description or diagnosis; later, the name *G.paniculata* was validly described by Choisy (1849). The name *G.paniculata* has been lectotypified in a first-step by Maheshwari (1964), who cited “Type: ex Sylhet, E. Pakistan, cult. in Indian Botanic Garden, Calcutta”, without citing a specimen or herbarium and in a second-step by Mohanan et al. (2023) using the specimen *Wallich 4857*, noted locality as “HBC (Calcutta Herbarium)” at CAL [CAL0000065167], with an isolectotype at K [K001104077]. However, we located the specimen *Wallich Cat. 4857* representing three different materials, which are distinguished by *4857A*, *4857B* and *4857C* (in The Wallich Catalogue). The specimen *Wallich Cat. 4857A* (K [K001104076]) is from Calcutta Botanical Garden (H.B.C.), Buchanan-Hamilton’s Herbarium; *Wallich Cat. 4857B* (CAL [CAL0000065167], G [G00726286], K [K001104077]) is from H.B.C.; and *Wallich Cat. 4857C* (BR [BR0000036486724], G [G00726273] and K [K001104078]) is from Sylhet (“Silhet” or “Sillet”) and, following Art. 9.6 of the ICN (Turland et al. 2018), these are syntypes. We think Maheshwari’s and Mohanan et al.’s typifications are mistaken. Since the name *G.paniculata* was validly described by Choisy (1849), a Swiss botanist working in Geneve, it is more plausible that he analysed material from G. Therefore, the specimen *Wallich Cat. 4857B* at G [G00726286], is selected here as the lectotype, with isolectotypes at CAL [CAL0000065167] and K [K001104077], following Art. 9.3 and 9.12 of the ICN (Turland et al. 2018).

*Garciniarhumicowa* was named by Jacques Denys Choisy, based on the specimen *Wallich Cat. 4858* collected from Calcutta Botanical Garden (H.B.C.) and Sylhet (“Sillet”) (Choisy 1849). We located the specimen *Wallich Cat. 4858* which represents two different materials collected from two different localities and which are distinguished by *4858A* and *4858B* (in The Wallich Catalogue). The specimen *Wallich Cat. 4858A* (CAL [CAL0000065164, CAL0000065168], K [K001104079]) is from Calcutta Botanical Garden and *Wallich Cat. 4858B* (BR [BR0000036486748], CAL [CAL0000065165], G [G00726295], K [K000677604, K001104080], P [P04701880, P04701886]) is from Sylhet and, following Art. 9.6 of the ICN (Turland et al. 2018), these are syntypes. It is more plausible that Jacques Denys Choisy (1799–1859), a Swiss botanist working in Geneve, analysed material from G. The specimen *Wallich Cat. 4858B* at G [G00726295] should be considered as a lectotype, with isolectotypes at BR [BR0000036486748], CAL [CAL0000065165], K [K000677604, K001104080] and P [P04701880, P04701886], following Art. 9.3 and 9.12 of the ICN (Turland et al. 2018).

*Garciniamckeaniana* was described by William Grant Craib, who cited two gatherings, *A. F. G. Kerr 3470* and *A. F. G. Kerr 3504* collected from Doi Suthep, at elevations of 1,200–1,550 m a.m.s.l. (Craib 1924). He did not mention the name of the herbaria where the materials were housed and, following Art. 9.6 of the ICN (Turland et al. 2018), these are syntypes. We located the materials *A. F. G. Kerr 3470* (1,200 m a.m.s.l.) at BM [BM000611632], K [K000677701] and P [P05061534] and *A. F. G. Kerr 3504* (1,550 m a.m.s.l.) at BM [BM000611633] and K [K000677702]. The material *A. F. G. Kerr 3470* at K [K000677701] is better preserved and more complete than the others and is designated here as the lectotype, with isolectotypes at BM [BM000611632] and P [P05061534], following Arts. 9.3 and 9.12 of the ICN (Turland et al. 2018).

##### Notes.

According to previous studies (e.g. Anderson (1874); Kurz (1877); Craib (1924); Gagnepain (1943); Maheshwari (1964); Mabberley (1977); Jones (1980); Long (1984); Singh (1993)) and based on the specimens that we examined, *Garciniamckeaniana* and *G.sopsopia* are similar and the vegetative and reproductive characters are overlapping between the two taxa. Therefore, *G.mckeaniana* is not morphologically distinguishable from *G.sopsopia* and is treated here as a new synonym.

*Garciniasopsopia* is recognised by its staminate flowers in terminal, much-branched thyrses with many to numerous flowers; pistillate flowers in terminal, short-branched thyrses (raceme-like), fewer in number of flowers than staminate; tetramerous flowers; numerous stamens (in staminate flowers) united into a single central 4-sided or weakly 4-lobed bundle surrounding a pistillode; the leaves with scalariform tertiary veins; and characters of fruits.

A comparison of morphological characters of *Garciniasopsopia* in Thailand with previous studies is summarised in Table 1.

**Table 1.** A comparison of morphological characters of *Garciniasopsopia* in Thailand with previous studies.

Characters	In this study	Previous studies
Position of staminate inflorescences	Terminal in agreement with Craib (1924), Kanjilal et al. (1934), Gagnepain (1943), Maheshwari (1964), Jones (1980) and Long (1984)	Axillary (Wight 1838)
Pistillate flowers	In a short-branched thyrse and the number of pistillate flowers in each inflorescence is fewer than staminate flowers in agreement with Jones (1980)	In a spike (Roxburgh 1832; Brandis 1906), in a short few-flowered, spike-like raceme (spicate raceme), rarely branched (Kurz 1877; Kanjilal et al. 1934; Maheshwari 1964; Singh 1993) or in a raceme (Long 1984)
Colour of flowers	Yellow in staminate and pistillate flowers	Pure or dull white (Anderson 1874; Brandis 1906; Maheshwari 1964; Singh 1993) or white in staminate flowers and yellow in pistillate flowers (Jones 1980)
Pistillode	Present in agreement with Gagnepain (1943)	Absent (Jones 1980; Singh 1993)
Number of seeds per fruit	3–7	3–5 (Maheshwari 1964; Singh 1993) or 4 (Anderson 1874; Kurz 1877; Kanjilal et al. 1934)

##### Additional specimens examined.

**Thailand. Northern.** • Chiang Mai [Doi Suthep, ♂ fl., 2 Jan 1915, *A. F. G. Kerr 3504* (BM [BM000611633], K [K000677702]); • ibid., fl., 6 Oct 1958 (as *Garcinia* sp.), *T. Sorensen et al. 5492* (C); • ibid., ♂ fl., 26 Dec 1987 (as *G.mckeaniana*), *J. F. Maxwell 87-1648* (L [L2416545]); • Doi Suthep-Pui National Park, fr., 19 Apr 1991 (as *G.mckeaniana*), *J. F. Maxwell 91-361* (AAU, A [GH00429134], P [P05061535]); • Huai Khok Ma, Doi Suthep-Pui National Park, fr., 8 Jun 1995 (as *G.mckeaniana*), *S. Kopachon S128* (CMUB); • ibid., 18 Jun 2003 (as *G.mckeaniana*), *J. F. Maxwell et al. 4* (CMUB); • Doi Suthep-Pui National Park, between Doi Suthep Temple and Chang Khian Valley, ♂ fl., 9 Oct 1997 (as G.aff.propinqua), *P. Sidisunthorn & S. Gardner 2371* (CMUB); • near Wat Phra That Doi Suthep, Doi Suthep-Pui National Park, ♂ fl., 29 Sep 2013 (as *G.mckeaniana*), *Tong Lau 1* (CMUB); Khun Chang Khian, Mueang District, ♂ fl., 29 Oct 1994 (as *Garcinia* sp.), *BGO. Staff 2456* (QBG); • ibid., ♂ fl., 29 Oct 1994 (as *Garcinia* sp.), *W. Nanakorn et al. 2479* (*BGO. Staff 2479*) (AAU, QBG); Doi Angka, Mae Ka Pak drainage, ♂ fl., 18 Nov 1930 (as *G.mckeaniana*), *H. B. G. Garrett 607* (BKF, C, K, L [L2416546]); • Doi Inthanon, fr., 21 Mar 1996 (as *Garcinia* sp.), *BGO. Staff 6204* (QBG); • Huai Sai Lueang Waterfall, Doi Inthanon, fr., 22 Mar 2002 (as *Garcinia* sp.), *T. Wongprasert et al. 023-37* (BKF); • Doi Inthanon, Mae Chaem Distritct, along stream, near Huai Sai Lueang Waterfall, at an elevation of 1,060 m a.m.s.l., fr., 24 May 2023, *C. Ngernsaengsaruay & T. Kaewgrajang G57-24022023* (BKF)]; • Nan [Hue Wao, fr., 10 March 1921 (as *G.mckeaniana*), *A. F. G. Kerr 5065* (BKF, BM, K); • Doi Phu Kha National Park, ♂ fl., 13 Jan 2000, *P. Srisanga 1275* [AAU & QBG (as *Garcinia* sp.), BKF & CMUB (as *G.pedunculata*)]; • ibid., fr., 27 May 2000 (as *G.pedunculata*), *P. Srisanga 1481* (QBG); •Tham Sakoen National Park, Yot Subdistrict, Song Khwae District, fl., 16 Dec 2010 (as *G.pedunculata*), *W. La-ongsri & N. Romkham 1282* (QBG)]; • Phrae [Mae Kray, ♂ fl., 10 Jan 1972 (as *G.mckeaniana*), *C. F. van Beusekom et al. 4788* (BKF, C, K, P [P05062052])]; • Uttaradit [Khao Phlueng, fl., 20 Dec 1943 (as *G.mckeaniana*), *P. Kanchanapan 30* (BKF)]; • Tak [Ler Tor Royal Project Area, Mae Ramat District, at an elevation of 1,250 m a.m.s.l., ♂ fl., 14 Dec 2024, *C. Ngernsaengsaruay et al. G58-14122024* (BKF)]; • Kamphaeng Phet [Khlong Lan, Mae Wong National Park, ♂ fl., 10 Oct 1999 (as *G.plena*), *M. van de Bult 380* (CMUB)]; **North-eastern.** • Loei [Lone Tae, Phu Luang Wildlife Sanctuary, fr., 17 May 1998 (as *Garcinia* sp.), *T. Wongprasert s.n.* (BKF124471); • ibid., fr., Aug 1998 (as *Garcinia* sp.), *T. Wongprasert s.n.* (BKF126762)].

**India.** • Cultivated in Calcutta Botanical Garden (H.B.C.), ♂ fl., 31 Dec 1814 (as *G.paniculata*), *Wall. Cat. 4857A* (Buchanan-Hamilton’s Herbarium) (K-W [K001104076]); • ibid., ♂ fl., s.d. (as *G.bhumicowa*), *Wallich Cat. 4858A* (CAL [CAL0000065164, CAL0000065168], K-W [K001104079]); • ibid., fl., s.d. (as *G.bhumicowa*), *Unknown s.n.* (E [E00839542]); ibid., fl., Dec 1814 (as *G.paniculata*), *F. Buchanan-Hamilton 1022* (E [E00839543]); • ibid., ♂ fl., s.d. (as *G.paniculata*), *Unknown s.n.* (K [K000677603], L [L2417597], P [P04701882]); • Assam, ♂ fl., 1863 (as *G.paniculata*), *C. Jenkins* (*Herb. L. Pierre 4578*) (P [P04701879, P04701887]); • ibid., s.d. (as *G.paniculata*), *C. Jenkins s.n.* (P [P04701884, P04701885]); • ibid., ♂ fl., 1865 (as *G.paniculata*), *C. Jenkins s.n.* (G [G00726260]); • Meghalaya, (East) Khasi Hills, Cherrapunjee, ♀ fl., 24 Jul 1952 (as *G.paniculata*), *W. N. Koelz 30814* (L [L2417594], MICH [1507203]); • Khasia, Regio trop., young fr., 4 Dec 1850 (as *G.paniculata*), *J. D. Hooker & T. Thomson s.n.* (K [K003668822]); • ibid., young fr., s.d. (as *G.paniculata*), *J. D. Hooker & T. Thomson s.n.* (G [G00726242], L [L2417595, L2417596], P [P04701883]); • Khasia, Regio trop, Churra, young fr., 16 Jun 1850 (as *G.paniculata*), *J. D. Hooker & T. Thomson 943* (K [K003668814]); East India, ♂ fl., s.d. (as *G.paniculata*), *W. Roxburgh s.n.* (BM [BM000611602], K [K000677602]); • Garo Hills, Tura Mountain, fl., s.d. (as *G.paniculata*), *N. E. Parry 881* (K [K003668815]).

**Nepal.** • Locality unspecified, fl., s.d. (as *G.paniculata*), *N. Wallich s.n.* (CAL [CAL0000065163]).

**Bhutan.** • Sarbhang District, Burborte Khola near Phipsoo, young fr., 18 Mar 1982, *A. J. C. Grierson & D. G. Long 3845* (E [E00170196], K [K001331949]); • Gaylegphug District, Lodrai Khola near Gaylegphug, 21 Mar 1982, *A. J. C. Grierson & D. G. Long 3887* (E [E00170197], K [K003668996]).

**Bangladesh.** • Sylhet, ♂ fl., s.d. (as *G.paniculata*), *Wall. Cat. 4857C* (BR [BR0000036486724], K-W [K001104078]); Chittagong, fl., 1874 (as *G.paniculata*), *W. Schleich s.n.* (K [K003668821]); • Chittagong Hill Tracts, fr., Mar 1880 (as *G.paniculata*), *J. S. Gamble 7800* (K [K003668823]); • Ponasari, Kelatuli, 2 Sep 1944 (as *G.paniculata*), *J. Sinclair 3717* (E [E00839545]); • Cultivated in East Bengal, fl., s.d. (as *G.paniculata*), *Herbarium of the late East India Company 852* (K [K003668818], P [P04701881]).

**Myanmar.** • Mon State, Amherst [Kyaikkhami] District, Dawna Range, Ta-Ok Plateau, fr., 23 Mar 1909 (as *G.cowa*), *J. H. Lace 4754* (E [E00839544]); • Kachin State, Myitkyina District, Nammina to Namma, fr., 7 Mar 1910 (as *G.paniculata*), *J. H. Lace 5172* (E [E00839546]); • Sandoway District, Arakan Yoma, fl., 17 Jan 1931 (as *G.cowa*), *Bals 11938* (K [K003668816]); • Locality unspecified, Feb 1872 (as *G.cowa*), Presented by *the Council of King’s College s.n.* (K [K003668817]).

**Vietnam.** • Tonkin, O. de Chapa, Quan Hóa District, Xinh mun, fl., 14 Aug 1926 (as *G.mckeaniana*), *M. E. Poilane 12929* (K, P [P05061533]).

**Laos.** • Xieng Khuang, fl., 18 Nov 1920 (as *G.mckeaniana*), *M. Poilane 2330* (K, P [P04899369]); • Khammouan, Nam Theun, Kaeng Luang, fl., 3 Nov 2005 (as *Garcinia* sp.), *M. F. Newman et al. LAO836* (BKF, L [L2409472, L2409473], P [P04897552]); • Khammouan, fl., 4 Nov 2005 (as *Garcinia* sp.), *M. F. Newman et al. LAO855* (BKF165806, BKF165964, BKF168376, L [L2409466, P04897550].

## Supplementary Material

XML Treatment for Garcinia sopsopia

## ﻿Additional information

### Author ORCIDs

Chatchai Ngernsaengsaruay https://orcid.org/0000-0002-7131-976X

Pichet Chanton https://orcid.org/0009-0001-7325-6109

Tharnrat Kaewgrajang https://orcid.org/0000-0001-7736-3596

### Data availability

All of the data that support the findings of this study are available in the main text.
